# Personal brain and Spatiotemporal Psychopathology - Intrinsic vs. extrinsic sources of interindividual variability

**DOI:** 10.1038/s41380-024-02783-3

**Published:** 2024-10-16

**Authors:** Georg Northoff, Jonas Daub, Dusan Hirjak

**Affiliations:** 1 Mind, Brain Imaging and Neuroethics Research Unit, The Royal’s Institute of Mental Health Research, University of Ottawa, Ottawa, ON Canada; 2 Department of Psychiatry and Psychotherapy, Central Institute of Mental Health, Medical Faculty Mannheim, University of Heidelberg, Mannheim, Germany; 3 German Centre for Mental Health (DZPG), Partner Site Mannheim, Mannheim, Germany

**Keywords:** Neuroscience, Diagnostic markers

In their impressive commentary, Stanghellini and Mecacci [1] pointed out one dimension which we neglected in our perspective [2], namely that the brain is intrinsically personal and thus individual. They cited unexpected sources like Iwan Petrowitsch Pawlow (1849–1936) and Alexander Romanowitsch Lurija (1902–1977) showing that even reflexes differ from individual to individual person. Obviously, we are confronted with an enormous interindividual variability in both brain data and subjective experience. Even the same symptom like sadness or delusion may be experienced in completely different ways by different subjects and may be manifest in different neural patterns [3, 4]. Hence, interindividual variability is a key issue in current scientific psychiatry which heavily contributes to its lack of translation into the clinical realm.

**Fig. 1:**
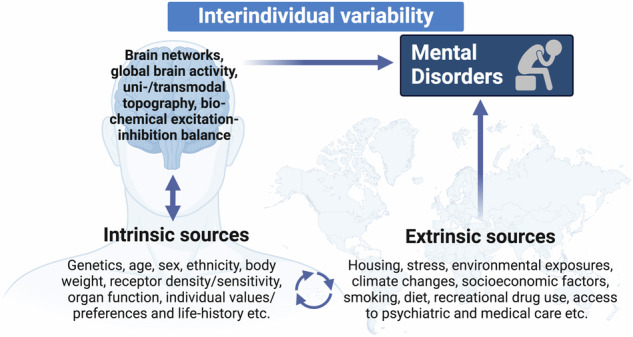
Intrinsic and extrinsic sources of interindividual variability contributing to mental disorders. This figure illustrates the interplay between intrinsic factors and extrinsic sources that contribute to interindividual variability. The figure highlights how the interaction between these intrinsic and extrinsic sources can increase susceptibility to various mental disorders, emphasizing the complex, multifactorial nature of mental health problems.

We were therefore asking ourselves where such interindividual variability comes from. Obvious sources discussed these days are low number of subjects and noise in the data themselves. Interindividual variability is here considered to be a merely extrinsic feature that remains different from the “true” or intrinsic features of the data. Large scale data sets providing “cleaner” data are the standard response to such extrinsic sources of intersubject variability [5]. In contrast, expanding upon the commentary by Stanghellini and Mecacci [1], we argue that interindividual variability is intrinsic as it relates to sources that define both brain and experience by themselves. This will ultimately affect the disease process itself in mental disorders which therefore, by their very nature, exhibit highly interindividual variability. We identify both intrinsic and extrinsic sources of interindividual variability, the brain itself and the environmental exposure (Fig. 1), which are closely interconnected.

Let us start with the environment. It is well-known by now that environmental events can trigger major neural and psychological changes [6–10]. There is no one-to-one relationship between environmental exposures (extrinsic sources) and neural changes (intrinsic sources), though. For instance, the same environmental event may be experienced as traumatic by one person and as trivial by another one. How is that possible? This is the moment where time and space come in.

Wang et al. [9] investigated traumatic events, e.g., childhood traumatic questionnaire (CTQ), time perspective with the Zimbardo time perspective inventory (ZTPI), and depressive symptom severity, e.g., Beck depression inventory-II (BDI-II), in a large cohort of depressed subjects. Applying mediation models, they found that the subjects’ experience of their time perspective of past, future and present, as tested for in the ZTPI, mediates the relationship of CTQ with BDI-II amounting to a full mediation. While the reverse with depression severity as mediator and time perspective as dependent variable did not hold. This means that traumatic life events in the childhood affect depressive symptoms severity through the modulation by the subjects’ time experience of past, present and future.

Accordingly, it depends upon how you perceive time with respect to past and future and whether an event in your childhood makes you depressed or not. If you are more future-oriented, you will have lower depression severity even if you have high CTQ scores. While a more past-focused time perception will make high depression severity more likely. Accordingly, nothing goes without the experience of a time perspective of past, present and future which is the key feature for transforming life events into depression or not. Conceived in a larger scope, time and space perception changes are not a consequence of the psychopathological symptoms but rather their antecedents by shaping and modulating them. This is further supported by a recent study where we showed that altered spatiotemporal experiences in anxiety disorder are related to anxiety severity as mediated by thought racing [11]. Using again mediation models, we showed that the time-space experience of anxiety modulates anxiety severity, e.g., Beck anxiety inventory, through its effects on thoughts, e.g., mind wandering spontaneous and deliberate. As in our earlier case, the subjects’ experiences of time and space are here an antecedent to the psychopathological symptoms themselves, e.g., the anxiety; this supports their fundamental character as “basic disturbance”.

But where and how do these time space perception changes come from? This leads us to the brain’s intrinsic activity featured by its own spatial, e.g., topographic and temporal, e.g., dynamic, patterns as outlined in our target article [2]. These topographic and dynamic patterns of the brain’s intrinsic activity are highly variable between different individual subjects, *“no one brain is the same as another one”* [12]. They constitute the brain’s inner time and space which are shared with the phenomenal and, more specifically, the temporal and spatial features of our experience (“common currency”) [12]; the latter are consecutively by themselves highly variable between different subjects, *“no one experience is the same between subjects”* [12].

Further, the brain’s inner time and space are also shared, at least in part, with the temporal and spatial features of our external environment; this renders our intrinsic brain highly sensitive and vulnerable to the extrinsic environmental events [8]. We consequently assume that the link of extrinsic environmental events and the brain’s intrinsic spatiotemporal organization, e.g., its topography and dynamics, is one key source of the high interindividual variability in both brain data and subjective time and space experience.

There is yet another factor to be considered, though. This leads us to the question what role or function the brain’s spontaneous activity serves? We currently do not know. We suggest that the brain’s spontaneous activity, through its intrinsic topography and dynamics [13, 14], serve as an internal reference, standard or baseline for all subsequent neural and psychological activity including time and space experience. If, for instance, my brain’s intrinsic speed of its neural activity is too slow, I will experience even slower external events as too fast as these are compared with the brain’s intrinsic speed, its internal baseline, reference or standard [15]. Hence, we suggest that the brain’s internal baseline, standard or reference is intrinsically spatial and temporal and henceforth highly specific to me and my individuality. For that reason, such spatiotemporal baseline of the brain may mediate the large interindividual variability of both subjective experience and brain data. In clinical terms this insight is of high relevance. We primarily aim to improve in our treatment the individual person with her or his specific experiences rather than the underlying brain as such: *“we as clinical psychiatrists, do not usually sit in front of a broken brain – we sit in front of a suffering person”* [16]. Consequently, it is of paramount importance that physicians embed the diagnosis in the individual life history and lifeworld of the patient, as we have already argued elsewhere [17]. This task is in its essence a hermeneutic procedure, which focusses on the individual specifics instead of neglecting them. This amounts to a truly dynamic view of mental disorders as reflected in the following quote by Stanghellini: *“A different representation of illness is called “dynamic”. According to this view, illness is not an accident that arrives from outside and upsets the state of equilibrium of an otherwise healthy organism. Humans are intrinsically vulnerable beings who fall ill when they respond incongruously to what they perceive as a threat for the unstable and vulnerable equilibrium characterizing their condition. This threat must not necessarily be an objective noxious entity; it is enough that it is subjectively experienced as such“* [18].

In sum, we can only agree with Stanghellini and Mecacci [1] in their plea for a personal brain. The sources of interindividual variability are not only extrinsic but deeply intrinsic as related to both brain and experience. The brain is indeed intrinsically personal and that, as we here proposed, may be related to its intrinsic spatiotemporal pattern, its topography and dynamics, serving as internal baseline while, at the same time, being strongly shaped and influenced by external environmental events. Developing these highly personal and individual spatiotemporal features of both brain and experience will be key in making possible the translation of Spatiotemporal Psychopathology into the clinical realm of diagnosis and therapy.
